# Hand function in children with radial longitudinal deficiency

**DOI:** 10.1186/1471-2474-14-116

**Published:** 2013-03-28

**Authors:** Anna Gerber Ekblom, Lars B Dahlin, Hans-Eric Rosberg, Monica Wiig, Michael Werner, Marianne Arner

**Affiliations:** 1 Karolinska Institutet, Department of Clinical Science and Education, Södersjukhuset, Section of Hand Surgery, Stockholm, Sweden; 2 Department of Hand Surgery, Södersjukhuset, Stockholm, Sweden; 3 Department of Clinical Sciences Malmö, Section of Hand Surgery, Lund University, Malmö, Sweden; 4 Department of Hand Surgery, Skåne University Hospital, Malmö, Sweden; 5 Department of Surgical Science, Hand Surgery, Uppsala University, Uppsala, Sweden; 6 Department of Hand Surgery, Uppsala University Hospital, Uppsala, Sweden; 7 Department of Radiology, Södersjukhuset, Stockholm, Sweden

**Keywords:** Radial longitudinal deficiency, Aplasia of the radius, Radial aplasia, Radial club hand, Hand function, Children, Functional outcome, ICF-CY, AHA, CHEQ

## Abstract

**Background:**

In children with hypoplasia or aplasia of the radius (radial longitudinal deficiency) manual activity limitations may be caused by several factors; a short and bowed forearm, radial deviation of the wrist, a non-functional or absent thumb, limited range of motion in the fingers and impaired grip strength. The present study investigates the relation between these variables and activity and participation in children with radial dysplasia.

**Methods:**

Twenty children, age 4–17 years, with radial longitudinal dysplasia Bayne type II-IV were examined with focus on the International Classification of Functioning and Health, version for Children and Youth (ICF-CY) context. *Body function/structure* was evaluated by measures of range of motion, grip strength, sensibility and radiographic parameters. *Activity* was examined by Box and Block Test and Assisting Hand Assessment (AHA). *Participation* was assessed by Children’s Hand-use Experience Questionnaire (CHEQ). Statistical correlations between assessments of body function/structure and activity as well as participation were examined.

**Results:**

The mean total active motion of wrist (49.6°) and digits (447°) were less than norms. The mean hand forearm angle was 34° radially. Ulnar length ranged from 40 to 80% of age-related norms. Grip strength (mean 2.7 kg) and Box and Block Test (mean 33.8 blocks/minute) were considerably lower than for age-related norms. The mean score for the AHA was 55.9 and for CHEQ *Grasp efficiency* 69.3. The AHA had significant relationship with the total range of motion of digits (*p* = 0.042). Self-experienced time of performance (CHEQ *Time*) had significant relationship with total active motion of wrist (*p* = 0.043). Hand forearm angle did not show any significant relationship with Box and Block Test, AHA or CHEQ.

**Conclusion:**

In radial longitudinal deficiency total range of motion of digits and wrist may be of more cardinal importance to the child’s activity and participation than the angulation of the wrist.

## Background

Radial longitudinal deficiency (RLD) is a rare congenital condition (0.2-0.5/10 000 live births) [1-4], with varying underdevelopment of the radial side structures of the upper limb, ranging from very mild thumb hypoplasia to complete absence of the radius. The more pronounced anomalies are characterized by a short and radially bowed forearm, radial deviation of the wrist, stiff fingers and a sub-functional or absent thumb. In these individuals, not only the skeletal structures are anomalous, but also muscles, tendons, vessels and nerves on the radial side of the forearm and hand. Children with RLD are bilaterally affected in more than half of the cases, but the severity of the deformity is often asymmetric [5]. Many of these individuals also have an associated non-hand anomaly and RLD is sometimes part of a known syndrome or a non-random association of congenital malformations (e.g. VATER) [5-10]. Surgical methods aim to improve function and appearance of the arm by correcting the angulation of the wrist and thereby also increasing the functional length of the limb and improving wrist position for later pollicization.

Previous studies have mainly focused on the results of surgery [6,11-15]. Several retrospective cases series have verified high rates of late deformity recurrence and significant impairment of ulnar growth after corrective surgery in RLD [12-14,16]. Since the long-term results are discouraging, it is of outmost importance to elucidate which component of the deformity that in fact is most essential for the child’s activity and participation. Few studies have evaluated hand function in individuals with RLD in terms of perceived disability and patient related outcome measures [5,17-19]. The focus of the present study was to investigate the relations between different components of the deformity and the activity and participation among individuals with RLD, regardless of type of deformity and prior treatment.

The ICF-CY (International Classification of Functioning and Health, version for Children and Youth, WHO 2007) gives a comprehensive description of disability and therefore we have adopted this framework in the present study.

Our hypothesis, to our knowledge not previously investigated, was that radial angulation may not be the primary determinant for the child’s activity and participation, nor for the self-rated opinion of appearance.

## Methods

This study is a Swedish multicenter study with the cooperation between the departments of Hand Surgery at Södersjukhuset, Stockholm, Uppsala University Hospital, University Hospital of Linköping, and Skåne University Hospital Malmö. All children with a diagnosis of RLD treated at these four departments were identified in medical registers. We obtained mandatory approval for the study from the Regional Ethical Review Board in Stockholm (ethical permit number 2010/1125-31/3, 2011/626-32).

The inclusion criteria were children age 4–17 years and unilateral or bilateral RLD Bayne type II-IV [6,20] (Table 1). Thirty-one children in the medical registers fulfilled the inclusion criteria. Out of these, one was deceased and one lived abroad. The parents of the 29 remaining children were informed of the purpose of the trial and asked to enrol their child in the study. Three families did not respond and six families declined participation due to lack of time or the child having an additional severe disease. Out of these nine children, six were boys and three girls between 7 and 16 years. Eight out of these children had a RLD Bayne IV and one child Bayne II. Twenty families gave their signed informed consent for participation.

**Table 1:** Modified Bayne classification of RLD

***Type***	***Thumb***	***Carpus***	***Distal radius***	***Proximal radius***
N	Hypoplastic or absent	Normal	Normal radius	Normal
0	Hypoplastic or absent	Absence, hypolasia or coalition	Normal radius	Normal, radio-ulnar synostosis or congenital dislocation of radial head
I	Hypoplastic or absent	Absence, hypolasia or coalition	Radius > 2 mm shorter than ulna	Normal, radio-ulnar synostosis or congenital dislocation of radial head
II	Hypoplastic or absent	Absence, hypolasia or coalition	Hypoplasia	Hypoplasia
III	Hypoplastic or absent	Absence, hypolasia or coalition	Partial absence of the radius	Variable hypoplasia
IV	Hypoplastic or absent	Absence, hypolasia or coalition	Absent	Absent

**Figure 1:**
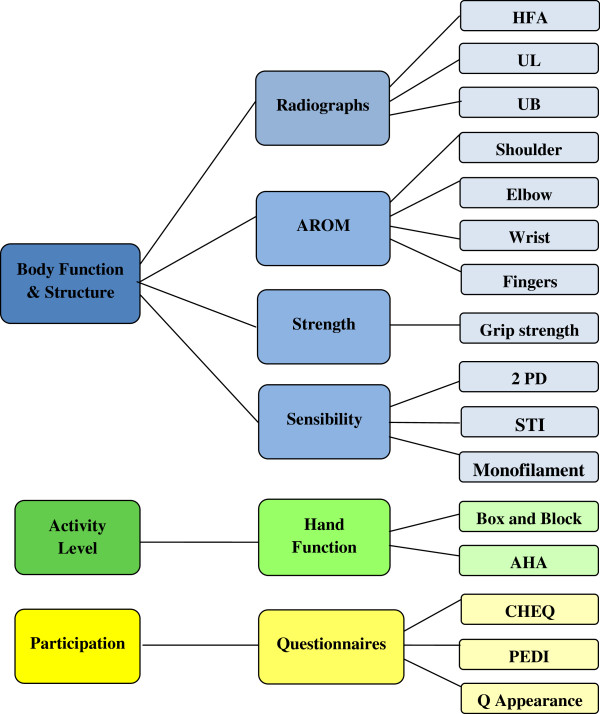
**Study Design ICF-CY Method.** Abbreviations: HFA (Hand forearm angle), UL (Ulnar length), UB (Ulnar bow), AROM (Active range of motion), 2-PD (Two-point discrimination test), STI (Shape-texture- identification test), AHA (Assisting hand assessment), CHEQ (Children’s hand-use experience questionnaire, PEDI (Pediatric evaluation of disability inventory), Q (Questionnaire).

The design of the study was in the ICF-CY context covering three different aspects of functioning; body function and structure, activity and participation (Figure 1).

### Body function and structure - objective assessment

All children were examined by either of two paediatric hand surgeons (AGE, HER). Active range of motion (AROM) was measured with a handheld goniometer for shoulder, elbow, wrist, metacarpophalangeal (MCP) joints, proximal interphalangeal joints (PIP), distal interphalangeal (DIP) joints and thumb interphalangeal joint (IP) on both sides. Total active motion of elbow (TAM Elbow) and all digits (TAM Digits), defined as the sum of AROM in MCP, PIP and DIP and IP joints, as well as arc of wrist extension to flexion (TAM Wrist Ext-Flex) were calculated. All measurements of AROM for shoulder, elbow, wrist and fingers were transformed to the Vilkki Severity Grading for RLD (HWO) [21], which takes in consideration mobility of the shoulder, elbow, wrist and fingers as well as thumb function. In addition, we also used a modification of the Vilkki Severity Grading for RLD (mH), which only includes the mobility of fingers and thumb (Table 2). The higher the score, the more severely affected limb: thus −1 being the best score. Grip strength was measured with an electronic Jamar dynamometer (E-Link®, Biometrics). This electronic device allows measurement at close to end range. During the measurements the child was seated with elbow flexed without resting the arm or the device on the table. The mean of three maximum voluntary contractions was recorded. All measurements were in kilograms (kg). Body length was measured in centimetres (cm). Sensibility of the radial and ulnar side of the thumb and each finger was examined with two-point discrimination test (2-PD) [22], Shape-Texture-Identification test (STI) [23] and Semmes-Weinstein monofilament test [24].

**Table 2:** Vilkki HWO severity grading for radial dysplasia

		***Severity points *HWO (−1 to 16p)**	***Severity points *mH (−1 to 9p*)***
**H**	**HAND (0-8p)**		
	MCP II-V flexion <45°	1/ digit	1/digit
	PIP II-V extension deficit >20°	1/digit	1/digit
**W**	**WRIST Radial deviation (0-4p)**		
	Mild 10-30°	1	
	Moderate 30-60°	2	
	Severe 60-90°	3	
	Extreme >90°	4	
**O**	**OTHER (0-3p)**		
	Elbow TAM <60° or weak flexion	1	
	Elbow Extension deficit >20°	1	
	Shoulder Abduction or Flexion <120°	1	
	Pinching pattern dig IV-V	1	1
	Functional or reconstructable thumb	−1	−1
	**Sum** Maximum	**16**	**9**

### Radiographic assessment

**Figure 2:**
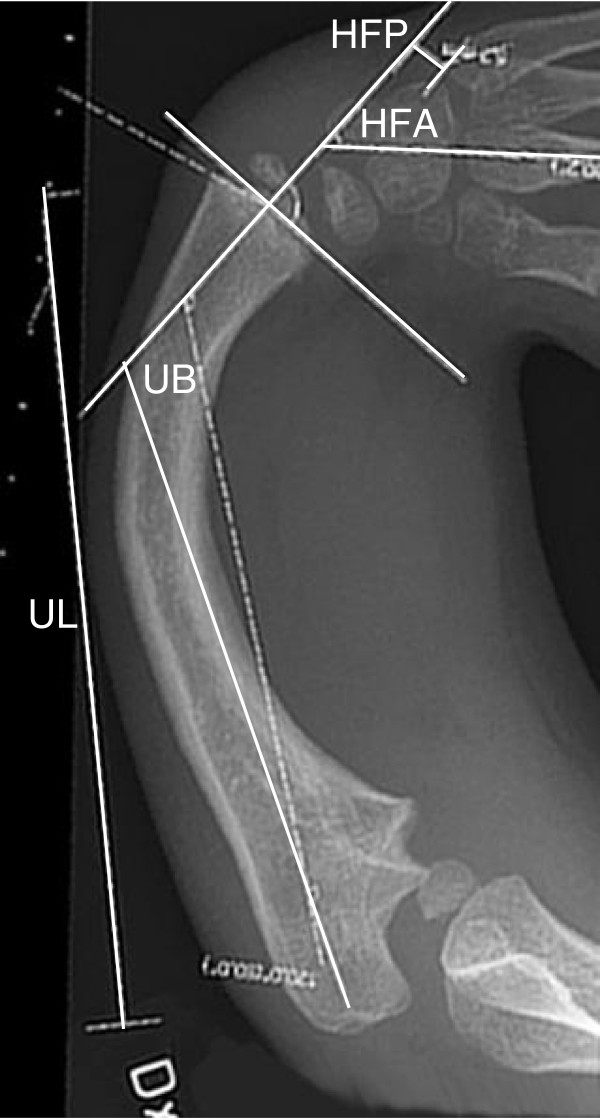
**Radiographic measurements.** Radiographic measurements were performed on the postero-anterior view of the forearm and hand. HFA (Hand Forearm Angle), HFP (Hand Forearm Position), UL (Ulnar Length), UB (Ulnar Bow).

Standard postero-anterior (PA) and lateral radiographs of arm, forearm, wrist and hand were taken bilaterally. The wrist was, without stress, positioned as straight as possible. All examinations and measurements (summarized in Figure 2.) were analyzed by one radiologist (MWe) specialized on the upper limb. To standardize the radiographic measurements and calculations, the three radiographic parameters proposed by Manske et al. [25] were used. The hand-forearm-angle (HFA) is defined as the acute intersecting angle between the longitudinal axis of the third metacarpal and the longitudinal axis of the distal ulna. The hand-forearm-position (HFP) is defined as the shortest distance between a line drawn through the longitudinal axis of the distal ulna and the base of the ulnarmost metacarpal. Negative measurements (−mm) indicate radial transposition of the carpus and positive measurements indicate ulnar transposition. Ulnar bow is defined as the acute intersecting angle between a line through the longitudinal axis of the distal ulna and a line drawn through the longitudinal axis of the proximal ulna. Ulnar length (UL) was measured at the PA view between the midpoint of the visible distal and proximal ulna. In the younger children, measurements were made between the epiphyseal plates, and in the older children the measurements included the epiphyses [26] (Figure 2).

### Activity - functional outcome tests

Either of two occupational therapists (BW, KS) administered the functional tests. All children performed both Box and Block Test [27] and Assisting Hand Assessment (AHA) [28,29].

The Box and Block Test of Manual Dexterity is a tool for testing manual dexterity that has shown good validity and reliability. Normative data is available for ages 6–19 years. It consists of a wooden box, divided into two equal compartments by a 15.2 cm high partition. The child is instructed to transfer as many 2.5 cm cubes as possible from one compartment to another in one minute. The score for each hand is equal to the number of transferred cubes.

The AHA is a test initially developed for children with unilateral upper limb dysfunction from cerebral palsy or brachial plexus birth palsy. We used the new version, adjusted for individuals with reduction deficiencies of the upper limb, called the AHA-PAD (Prosthesis, Amputation, Deficiency), which is currently undergoing validation (personal communication L. Krumlinde-Sundholm 2012). In the AHA, the child is given toys which require the use of two hands and the efficiency of the affected hand, when spontaneously used in bimanual activity, is scored from the video recorded play session. In the research version of the AHA-PAD, 18 items consisting of observable actions have shown evidence of internal construct validity by adequate fit to the Rasch measurement model. Each item is scored on a 4-point rating scale assessing the quality of the performance. The raw sum score range from 18 to 72, with a higher number indicating higher ability. The sum scores are by the Rasch analysis, transformed to internal level logit measures, ranging from 0 to 100 AHA-units, where 100 is the maximal result. Since the AHA is a test designed for unilateral disorders, only the 15 children with unilateral RLD were included in the statistical analyses.

### Participation - subjective assessment

Children’s Hand-use Experience Questionnaire (CHEQ; www. cheq.se, Swedish version) [30], is a newly developed, web-based, validated questionnaire for evaluation of children’s experience of their performance in the disabled hand while doing bimanual tasks. The questionnaire includes 29 different activities, each rated on three scales covering different aspects of bimanual hand use; efficiency of the grasp, the time it takes to perform the task and whether the child feels bothered while doing it. In CHEQ, the raw score for the three different aspects of bimanual hand use can range from 1 to 4; the latter being the best score. By Rasch analysis [31], the raw sum scores are transformed to scaled scores ranging from 0 to 100, where 100 is the best. All children answered the CHEQ, either by themselves or with a parent assisting. Bilaterally affected children were informed to answer the questionnaire regarding their most severely affected hand. The CHEQ scaled score was considered to be the same for both arms in bilaterally affected children.

The Pediatric Evaluation of Disability Inventory (PEDI) [32,33] is a validated questionnaire for children with functional disabilities designed to evaluate the child’s performance and participation. The child’s parents answered questions in the domain Functional skills in PEDI. PEDI is validated for children aged 2.0-6.9 years or older children, if their level of function is below that of a non-disabled 7.5-year-old child. Since this study includes children up to age 17 years, only analysis of the specific tasks in PEDI were undertaken.

In addition to CHEQ and PEDI, all children, in some cases assisted by a parent, answered a short questionnaire where they could evaluate the appearance of the disabled hand(s) and arm(s) on a scale ranging from 1–5, the lower the better. This value was then transformed to a score out of 100 by subtracting one and multiplying by 25 (equivalent to scoring the Quick DASH(Disabilities of the Arm Shoulder and Hand) [34]). This transformation was done to make the score easier to compare to other measures scaled on a 0–100 scale. A lower score indicates greater satisfaction with the appearance of the arm(s).

### Statistical analysis

Descriptive statistics were presented for age, gender, CHEQ and appearance (n = 20, each individual), AHA (n = 15, unilateral cases only) and of affected side, grip strength, HFA, HFP, UL/BL%, UB, TAM Wrist Ext-Flex and TAM Digits (n = 25, each affected limb). Linear regression, adjusting for age, gender, side, uni-or bilateral affection and normal other side, was performed to test for association between Box and Block Test as well as the CHEQ questionnaire and the other variables, i.e. grip strength, HFA, HWO , mH, UL/BL-percent, UB, TAM Elbow, TAM Wrist Ext-Flex and TAM Digits, respectively. Linear regression, adjusting for age, gender, side and normal other side, was performed to test for association between the AHA and the other variables. The standardized regression coefficients are presented. The sandwich estimator was applied to Box and Block Test and to the CHEQ variables to correct for possible correlation due to the fact that five bilateral children were included twice. The normality assumption was assessed for the multiple linear regressions with QQ-plots and the Shapiro-Wilk test and no violations were detected for Box and Block Test, CHEQ *Grasp Efficiency* and CHEQ *Bother*. However, CHEQ *Time* showed signs of not following a normal distribution, but was for comparative reasons still analyzed with parametric methods. Thus, the result should be interpreted with care. All analyses were performed in R v 2.14.1 (R Foundation for Statistical Computing, Vienna, Austria) (regressions), IBM SPSS Statistics 20 (descriptive statistics) and Microsoft Office Excel 2007 (plots). The level of significance was set to 0.05 (two-sided).

## Results

### Demographics

**Figure 3:**
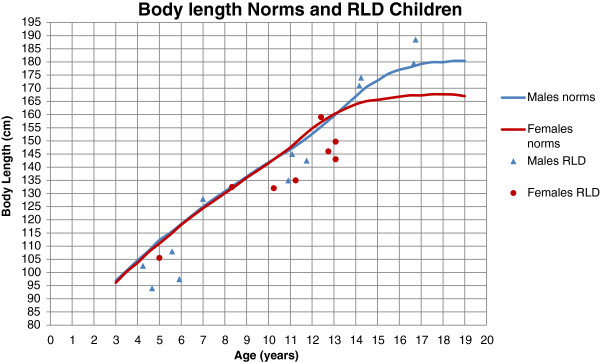
**Body length norms**[35]**and RLD children.**

General demographic data are presented in Table 3. The mean age of all 20 children was 10.5 years (median 11.1, range 4.3-16.8). Twelve children were boys and eight were girls. The children’s body length (BL) compared to Swedish norms [35] is presented in Figure 3. Seven of 20 children had a known general syndrome (Table 3). Five children had a bilateral RLD where both arms fulfilled the inclusion criteria. Eight children had less pronounced radial deficiency (Bayne 0 to I) on the not included side and seven children had a completely normal other side. There was preponderance of RLD for the left side (15/25 limbs). Bayne IV was the most common category (16/25) followed by Bayne II (5/25), and Bayne III was found in 4/25 limbs. In 18/25 limbs, surgical wrist correction had been performed, whereof 13 with prior soft tissue distraction Twelve of 25 limbs had been operated by radialization procedure [16], 6/25 by a non-notched centralization procedure [36] and three had undergone ulnar lengthening by callus distraction. Eleven of 25 hands had had a pollicization. Six limbs in five children were not surgically treated. Seven of the 15 unilateral cases had a completely normal opposite limb, but the remaining eight children had a varying degree of radial side affliction with carpal anomalies and thumb hypoplasia (Table 3).

**Table 3:** RLD children demographics

					**Classification**		**Vilkki severity grading classification for RLD**		**Range of motion**		**Radiographs**					**Treatment**					**Opposite hand if**	
**Child no**	**Gender**	**Age (yr)**	**Syndrome**	**Side**	**Bayne**	**Blauth**	**HWO**	**mH**	**Wrist Ext-Flex(°)**	**Digits TAM(°)**	**HFA(°)**	**UL(mm)**	**HFP(mm)**	**UB(°)93**	**BL(cm)**	**R/C**	**SD**	**CD**	**P/O/S/Rot**	**Nonsurgically**	**Bayne**	**Blauth**
1	F	5,0	Goldenhar	R	III	V	10	8	35	375	5	84	9.4	93	105.5	R	SD		P		None	None
2	F	8,3		L	III	IIIA	8	7	20	470	45.5	124	0	12	132.5	R	SD	CD	OS		None	None
3	M	5,9		L	IV	V	12	8	0	205	98	90	-18	6.5	97.5					NS	None	None
4	F	11,3		L	II	V	1	0	75	725	15.5	157	-8.5	4.5	135	R	SD		P		I	II
5	M	10,9	VATER	R	IV	IV	11	8	40	175	90.5	117	-11.5	37	135	R	SD		P		0	V
6	M	11,8		L	II	IIIA	4	3	85	730	20.5	158	-11	3	142.5			CD			0	II
7R	F	13,1		R	IV	V	13	9	0	70	24	96	8	52	143	C			P			
7 L	F			L	IV	V	9	5	95	145	65	123	-26	73	143					NS		
8	M	16,7		R	IV	V	12	8	20	145	*	108	*	61	179.5	C					None	None
9	F	12,8	VATER	L	IV	V	7	6	120	610	1	150	1.5	36	146	R	SD		P		I	II
10	M	14,3		R	III	V	8	5	55	290	-2	165	4	27	174	C					None	None
11	M	4,3		L	IV	V	12	6	60	315	79	86	-15	33	102.5		SD			NS	None	None
12R	F	13,1	TAR	R	IV	I	5	3	65	555	35	107	6.5	90	149.7	R						
12 L	F			L	IV	I	6	2	15	730	33	117	5.5	53	149.7	R	SD					III
13	F	12,4		L	III	V	11	8	55	475	17	175	3.5	45	159	R	SD		P		0	II
14R	M	5,6	VATER	R	IV	IV	12	8	30	125	42	104	-6.5	11	108	C						None
14 L	M			L	IV	V	12	8	75	285	76	99	-18	20	108		SD			NS		II
15	M	11,1	VATER	L	IV	V	9	7	40	310	63	137	-7.5	14	145	C	SD		Rot MCII		0	V
16	M	14,2		L	IV	V	11	6	0	355	29	147	0	71	171	R	SD	CD	P		None	
17	M	7,0		R	IV	IV	8	6	35	710	50	112	-5	60	128	R			P		0	
18	M	16,8	Goldenhar	L	IV	V	3	2	100	650	12	215	13.5	23	188.5	R			P		0	
19R	F	10,3		R	II	II	3	1	60	850	0	121	-5	30	132					NS		
19 L	F			L	II	II	-1	-1	95	1095	3	148	0	13	132					NS		
20R	M	4,7		R	II	IV	3	2	10	495	-7	111	6	25	94	C	SD		P			
20 L	M			L	IV	V	7	7	55	280	18	97	8	41	94	R	SD		P			

* = not measurable.

R = Radialization.

C = Centralization.

P = Pollicization.

O = Opponens plasty.

S = Stabilization.

Rot = Rotational osteotomy MCI.

SD = Soft tissue distraction.

CD = Callus distraction.

NS = Nonsurgical treatment.

None = normal.

Bayne 0 = carpal anomalies.

Bayne I = R < 2 mm shorter than U.

Bayne II = R hypoplastic.

Bayne III = R part absence.

Bayne IV = R absent.

### Radiographic assessment

**Figure 4:**
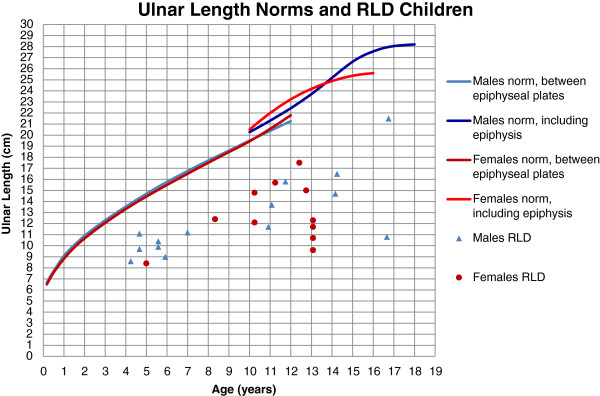
**Ulnar length norms**[26]**and RLD children.**

**Figure 5:**
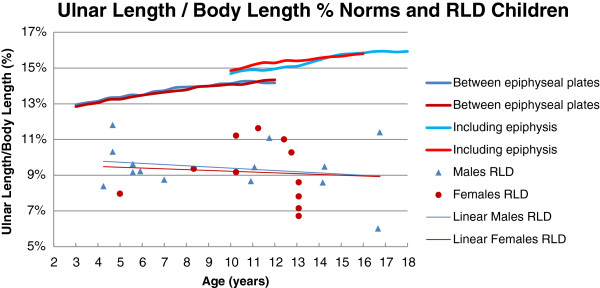
**UL/BL% norms**[26,35]**and RLD children.**

The results of radiographic measurements for each individual are shown in Table 3. The total HFA had a wide spread from -7° (ulnar deviation) to severe radial deviation of 98° (mean 34°, SD 31). In the non-surgically treated limbs, the HFA was greater (mean 54°, SD 42) than in the surgically treated limbs (mean 27°, SD 25). HFP, in relation to the longitudinal axis of the distal ulna, varied from radial transposition, -26 mm, to ulnar transposition, 18 mm, (mean 5.2 mm, SD 9.1). UB varied from 3° to 93° (mean 37°, SD 26). In the non-surgically treated limbs, the UB was less (mean 29°, SD 23) compared to the surgically treated limbs (40°, SD 27). UL compared to norms is presented in Figure 4. UL was markedly shorter, ranging from 40 to 80% of age-related norms. UL among the surgically treated limbs in this study was 62% of norms, which was equal to UL among non-surgically treated limbs. To get a comparable measure of UL regardless of the opposite limb, UL in relation to BL was calculated as UL percent of total BL (UL/BL%). The range of UL/BL% for the affected limbs was 6-12% (mean 9.3%, SD 1.5). In normally developed children aged 4–18 years, UL/BL% ranges from 13% for the younger children to 16% for the older [26]. On the contrary, UL/BL% among the children with RLD decreased with age (Figure 5).

### Functional outcome

The active range of motion of the shoulders was normal or close to normal in all limbs except in one child with bilateral RLD Bayne IV, who had restricted abduction and flexion in both shoulders. The TAM Elbow varied from 0° to 150° (mean 77.6°, SD 47.0). The TAM Wrist Ext-Flex spanned from 0° to 120° (mean 49.6°, SD 33.9) and the TAM Digits varied from 70° to 1095° (mean 447°, SD 263).

The mean HWO score was 7.8 (SD 3.9) and median 8.0 (range −1 to13). The mean mH score was 5.3 (SD 2.9) and median 6.0 (range −1 to 9). Six thumbs were considered to be functional or reconstructable. In twelve limbs, the pinching-pattern was between the two ulnarmost digits and in four of these hands the pattern was present despite former pollicization.

**Figure 6:**
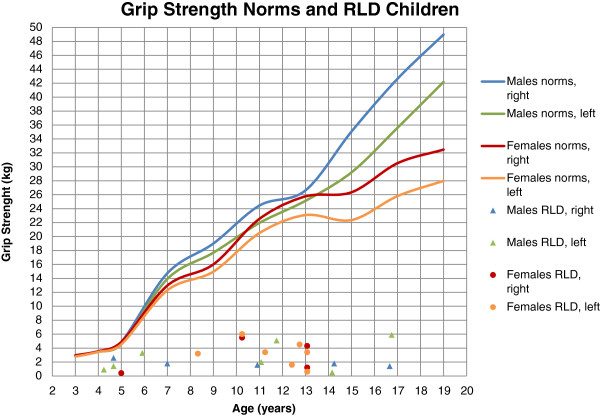
**Grip strength norms**[37]**and RLD children.**

One child with a bilateral RLD Bayne IV was unable to perform a power grip. Grip strength was considerably lower for the 23 measured limbs compared to norms [37] and did not increase with age as for normally developed children (Figure 6). Grip strength ranged from 0.4 to 6.0 kg (mean 2.7 kg, SD 1.8).

Sensibility, as tested with Semmes-Weinstein monofilament test, was normal in all hands, i.e. green filament (number 2.83) [38]. One child, with a bilateral RLD, Bayne IV and age 4 years, was not able to participate neither in the 2-PD test nor in the STI-test. 2-PD-test was normal (3-5 mm) [22] or close to normal (6 mm) in all 23 tested hands. The STI-scores were normal (6 points) [23] or close to normal (5 points) in 19/23 hands and subnormal (3–4 points) in 4/23 measured hands.

**Figure 7:**
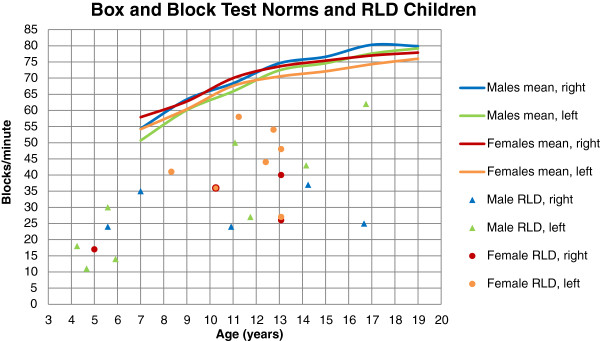
**Box and block norms**[27]**and RLD children.**

Box and Block Test values compared to norms [27] are presented in Figure 7. The results in the Box and Block Test for the children with RLD were considerably lower than norms. The number of blocks per minute varied from 11 to 63 (mean 33.8, SD 14.1).

The AHA scaled scores for the unilateral RLD children varied from 47 to 72 (mean 55.9, SD 6.0).

### Patient related outcome

Among the RLD children in this study, the mean score for CHEQ *Grasp efficiency* was 69.3 (SD 16.3), the mean score for the time it takes to perform the task (CHEQ *Time)* 63.3 (SD 19.5) and the mean score for whether the child feels bothered while doing it (CHEQ *Feeling* b*othered)* 70.5 (SD 18.1).

The most frequent tasks that were either impossible to perform or most difficult to perform, took longer time and bothered the children the most, were opening up a carton of milk or juice, cutting meat on a plate, fastening a necklace, tying shoelaces, peeling an orange, unscrewing the cap of unopened soft drink bottle, fastening a helmet and opening a bag (of e.g. crisps).

In PEDI Functional skills, self-care domain, 10/20 children had the highest scoring possible. For the remaining 10 children, the tasks they had most difficulties with were manipulation of fasteners and zippers in clothing, wiping self thoroughly after bowel movements and tying shoelaces.

The scores in the questionnaire regarding appearance of the arm(s) and hand(s) can range from 0 to 100, 0 being the best score. Despite of deformity, the children in this study gave fairly low scores (mean 26, SD 26, median 25, range 0–100).

### Statistical correlations

The statistical correlations are provided in Table 4. In the regression analyses the AHA score significantly correlated to HWO (p = 0.018) and TAM Digits (p = 0.042). CHEQ *Time* significantly correlated to TAM Wrist Ext-Flex (p = 0.043).

**Table 4:** RLD children statistical correlation

	**Box and Block**		**AHA**		**CHEQ *Grasp efficiency***		**CHEQ *Time***		**CHEQ *Feeling bothered***	
	***r***	***p***	***r***	***p***	***r***	***p***	***r***	***p***	***r***	***p***
Grip strength	0.22	0.211	0.57	0.146	0.08	0.735	−0.01	0.949	−0.13	0.408
HFA	−0.15	0.393	−0.94	0.055	−0.29	0.228	−0.37	0.140	−0.09	0.574
HWO	−0.21	0.210	−0.81	0.018	−0.15	0.531	−0.08	0.682	0.03	0.849
mH	−0.24	0.112	−0.57	0.091	−0.16	0.455	−0.07	0.705	0.03	0.887
UL/BL %	0.19	0.242	0.70	0.182	0.26	0.357	0.16	0.426	−0.03	0.893
UB	−0.15	0.314	−0.31	0.431	−0.22	0.269	−0.08	0.636	−0.03	0.842
TAM Elbow	0.08	0.604	0.46	0.201	−0.32	0.081	−0.22	0.138	−0.03	0.852
TAM Wrist Ext-Flex	−0.01	0.962	0.48	0.240	0.31	0.116	0.39	0.043	0.13	0.335
TAM Digits	0.27	0.137	0.78	0.042	0.24	0.207	0.18	0.244	−0.10	0.565

*p* = p-value, *r* = the standardized regression coefficient.

Multiple linear regression, adjusted for age, gender, side, uni-/bilateral and normal other side.

AHA not adjusted for uni-/bilateral.

## Discussion

The present study describes a small number of children with RLD, but with a wider perspective than commonly used since the different domains in the ICF-CY framework were used. Compared to normally developed children, the children with RLD had a considerable shortening of the forearm, angulation of the wrist and stiffness in the fingers as well as severely limited grip strength and low scores in manipulation tasks. Nevertheless, the performance in spontaneous bimanual activities was high, the self-perceived disability low and they esteemed the appearance of their arm high. Interestingly, the statistical analyses support our hypothesis that radial angulation of the wrist is of lesser importance for activity and participation than active wrist and finger motion.

In testing the hypothesis for this study, the diversity of clinical presentations in the children can be regarded more as a strength than as a weakness. The present study was neither comparing outcome after different surgical procedures nor relating severity grade and hand function, but instead investigated the relation between the different components of the deformity and activity and participation. The wide spread of clinical presentation facilitated this investigation.

In clinical evaluation of children with RLD it is important not only to focus on the anomaly per se, but also to assess possible participation restrictions for the child as well as the child’s own apprehension of the difficulties he or she meets in daily life. The ICF-CY context covers three different aspects of disability; *body function and structure*, *activity* and *participation*. Therefore the ICF-CY framework may better govern our treatment strategies.

We used two new outcome instruments for bimanual hand use in children, the AHA and the CHEQ. The AHA is currently under validation for reduction deficiencies and the new scoring system, AHA-PAD, was used in the present study. The CHEQ is a new questionnaire, which evaluates self-perceived disability in regards of grasp efficiency, time to complete tasks and bother. The Vilkki Severity Grading for RLD [21] and the present modification (mH), taking only the mobility of the fingers and thumb function in consideration, was an attempt to create a measure of the complex hand anomaly in RLD. The Vilkki score is, to our knowledge, not psychometrically evaluated, but gives a good impression of the extent of deformity. Since the AHA-PAD, CHEQ, HWO and mH were used on children with RLD for the first time in the present study, there are no available comparable data in the literature. However, examination of the correlations between *body function and structure* and *activity* as well as *participation* by the use of these new instruments, together with well-known measurements of deformity and function, e.g. AROM, radiographic measurements, grip strength, and Box and Block Test, are new important aspects in the ICF-CY framework to evaluate RLD.

UL is often discussed in relation to RLD. Even in the natural course of the anomaly, ulnar growth is markedly reduced and final ulnar length is approximately 1/2 – 3/4 of unaffected children [6,13,39]. Surgical treatment is considered to further reduce the ulnar growth [5,6,11,13]. The present children with RLD also had markedly reduced UL, i.e. 40-80% of norms [26] (Figure 4.). As opposed to other studies [11,13], UL among the present surgically treated limbs was similar to UL among non-surgically treated limbs. This is most likely due to 2/6 non-operated limbs being Bayne type II and all surgical procedures being without resection of carpal bones.

The HFA (34°) was greater than in the study of Goldfarb (25°) [17], which most likely is due to the fact that all limbs in that study were surgically treated by centralization, compared to the present study, where only 5/25 were treated by centralization and 13/25 limbs by radialization procedure.

The results from the assessments of the present children with RLD differed in several aspects from previous data. They had considerably lower grip strength (mean 2.7 kg; Figure 6.) compared to norms and also to those reported in other studies, (3.49 kg) [18] and (5 kg) [17], which may be explained by a difference in patient characteristics, i.e. inclusion of Bayne type I [18] and older patients [17]. However, since grip strength did not increase with age among the present children, the age difference is not a sufficient explanation.

TAM Digits (447°) is comparable to reported data in the study by Goldfarb et al. (110°/digit). Surprisingly, arc of wrist extension and flexion varies considerably between different studies, Buffart et al. (114°), Goldfarb et al. (31°) and the present study (50°). The explanation for this remains unclear, but might be due to different techniques when measuring range of motion as well as inclusion of Bayne type I patients in the study by Buffart et al. [18].

The children in the present study were more content with the appearance of their arm than the individuals in the study by Goldfarb et al. [17]. Individuals in the study of Goldfarb et al. had more severe types of RLD (Bayne III-IV) and all were surgically treated with centralization procedures. Furthermore, the individual’s apprehension of appearance of the anomalous limb may also differ because of varying sociocultural contexts.

We did not find any significant statistical correlations between HFA and the two functional tests, Box and Block test and AHA. In concordance, Goldfarb et al. [17] did not find any significant correlations between length of humerus, UL, HFA, HFP, resting angle of wrist or wrist arc of motion and the Jebsen-Taylor test or the DASH questionnaire.

In the present study, we only used tests and questionnaires developed for children. In line with our results, Buffart et al. [18] found significant correlations between AHA and grip strength, pinch strength, active range of motion the wrist and the second digit. However, in contrast to our study, the AHA used by Buffart et al. was the original version not validated for children with reduction deformities (AHA-PAD). They also included bilaterally affected children, which is not in line with the AHA instructions.

The children’s apprehension of time to perform different tasks (CHEQ *Time*) and TAM Wrist Ext-Flex showed significant correlation. This demonstrates the importance of mobility of the wrist for activity performance, which is in line with the statements by Buffart et al. [18] and Kotwal et al. [40].

We found no significant correlations between the children’s self-experienced grasp efficiency (CHEQ *Grasp efficiency)* and bother while doing them (CHEQ *Feeling bothered)* with any measures of body function and structure (Table 4). The CHEQ overall showed high scores, indicating that children with RLD, despite of the deformity, experience themselves as very able to perform bimanual tasks and, even though more time consuming, they do not feel considerably bothered. In the sub-analyses of different manual tasks in CHEQ and PEDI, the ones that were distinguished as difficult, were bimanual tasks requiring a strong grasp and pinch and prehensile skill.

The number of examined limbs in this study is small and therefore interpretations should be made with caution. The focus was not to compare surgical treatments, but to examine which parameters of body function and structure that were most important for activity and participation, as well as for self-perceived manual activity limitations and appearance. Comparison with previous studies could also be influenced by different radiographic projections, different methods of measuring range of motion and grip strength and different subjective evaluation of the child’s performance in the hand function tests by the occupational therapists. In this study we aimed to minimize the variations in radiographic measurements by using standardized methods [25] and having all radiographs examined by the same author. Furthermore, all measurements of range of motion were done by two of the authors in a standardized way and the functional tests were performed by only two occupational therapists.

The current surgical options in RLD are limited to correcting the radial deviation and enhancing grasp by pollicization. Improving active finger motion is not yet possible by surgical means. The notched centralization procedure has more negative impact on UL and wrist mobility than non-notched centralization and radialization [13,16]. The present results indicate AROM of wrist and digits are of more importance for functional outcome and self-experienced manual performance than the radial angulation of the wrist. In the future this should be considered when choosing treatment strategy. The surgical method that has the least negative influence on wrist mobility should be chosen. In some individuals a non-surgical treatment strategy may be a better option, still with a focus on maintenance of a good digital range of motion. Future studies should further investigate the relations between the different components of the complex malformation in RLD and hand function, especially in the long-term.

## Conclusions

We conclude that active range of motion in wrist and digits may be more important for activity and self-perceived disability in individuals with RLD than the radial angulation of the wrist. Future treatment regimes in RLD should perhaps strive more to increase or retain mobility of digits. When surgical correction of wrist angulation is performed, preserving wrist mobility may be most important for the individual. The self reported limitations of activity and participation as well as the apprehension of hand appearance are additional aspects of this complex anomaly that should be further studied.

## Abbreviations

AHA: Assisting hand assessment; AHA-PAD: Assisting hand assessment for prosthesis, amputation and deficiencies; AROM: Active range of motion; BL: Body length; CHEQ: Children hand-use experience questionnaire; HWO: Vilkki severity grading for radial dysplasia (Hand Wrist Other); HFA: Hand forearm angle; HFP: Hand forearm position; ICF-CY: International classification of functioning and health, version for children and youth, WHO 2007; mH: Modified vilkki severity grading for radial dysplasia (Hand); PEDI: Pediatric evaluation of disability inventory; RLD: Radial longitudinal deficiency; STI: Shape-texture-identification test; 2-PD: Two-point-discrimination test; TAM Digits: Total active range of motion of digits; TAM Elbow: Total active range of motion of elbow; TAM Wrist ext-flex: Total active range of motion of wrist, extension to flexion; UB: Ulnar bow; UL: Ulnar length.

## Competing interests

The authors declare that they have no competing interests.

## Authors’ contributions

AGE participated in the design of the study and in examination of the children, coordinated the study, participated in performing the statistical analyses and drafted the manuscript. HER and MWi participated in examination of the children and helped to draft the manuscript. MWe performed all radiographic assessments and measurements. LBD and MA participated in the design of the study and helped to draft the manuscript. All authors read and approved of the final manuscript.

## Pre-publication history

The pre-publication history for this paper can be accessed here:

http://www.biomedcentral.com/1471-2474/14/116/prepub
